# Expression of the Pluripotency Transcription Factor OCT4 in the Normal and Aberrant Mammary Gland

**DOI:** 10.3389/fonc.2013.00079

**Published:** 2013-04-11

**Authors:** Foteini Hassiotou, Anna R. Hepworth, Adriana S. Beltran, Michelle M. Mathews, Alison M. Stuebe, Peter E. Hartmann, Luis Filgueira, Pilar Blancafort

**Affiliations:** ^1^School of Chemistry and Biochemistry, Faculty of Science, The University of Western AustraliaPerth, WA, Australia; ^2^School of Anatomy, Physiology and Human Biology, Faculty of Science, The University of Western AustraliaPerth, WA, Australia; ^3^Department of Pharmacology, School of Medicine, The University of North CarolinaChapel Hill, NC, USA; ^4^Translational Pathology Laboratory, The University of North CarolinaChapel Hill, NC, USA; ^5^Department of Obstetrics and Gynecology, Division of Maternal-Fetal Medicine, School of Medicine, The University of North CarolinaChapel Hill, NC, USA; ^6^Anatomy Unit, Department of Medicine, University of FribourgFribourg, Switzerland

**Keywords:** mammary gland, breastmilk, breast cancer, transcription factors, OCT4, adult stem cell, cancer stem cell, self-renewal

## Abstract

Breast cancers with lactating features, some of which are associated with pregnancy and lactation, are often poorly differentiated, lack estrogen receptor, progesterone receptor, and HER2 expression and have high mortality. Very little is known about the molecular mechanisms that drive uncontrolled cell proliferation in these tumors and confer lactating features. We have recently reported expression of OCT4 and associated embryonic stem cell self-renewal genes in the normal lactating breast and breastmilk stem cells (hBSCs). This prompted us to examine OCT4 expression in breast cancers with lactating features and compare it with that observed during normal lactation, using rare specimens of human lactating breast. In accordance with previous literature, the normal resting breast (from non-pregnant, non-lactating women) showed minimal OCT4 nuclear expression (0.9%). However, this increased in the normal lactating breast (11.4%), with further increase in lactating adenomas, lactating carcinomas, and pregnancy-associated breast cancer (30.7–48.3%). OCT4 was expressed in the epithelium and at lower levels in the stroma, and was co-localized with NANOG. Comparison of normal non-tumorigenic hBSCs with OCT4-overexpressing tumorigenic breast cell lines (OTBCs) demonstrated upregulation of OCT4, SOX2, and NANOG in both systems, but OTBCs expressed OCT4 at significantly higher levels than SOX2 and NANOG. Similar to hBSCs, OTBCs displayed multi-lineage differentiation potential, including the ability to differentiate into functional lactocytes synthesizing milk proteins both *in vitro* and *in vivo*. Based on these findings, we propose a hypothesis of normal and malignant transformation in the breast, which centers on OCT4 and its associated gene network. Although minimal expression of these embryonic genes can be seen in the breast in its resting state throughout life, a controlled program of upregulation of this gene network may be a potential regulator of the normal remodeling of the breast toward a milk-secretory organ during pregnancy and lactation. Deregulation of this gene network either within or outside pregnancy and lactation may lead to aberrant breast cell proliferation and malignant transformation, suggesting a role of these genes in both normal lactation and breast oncogenesis.

## Introduction

Breast cancer associated with pregnancy and lactation (PABC, defined as concurrent with or within 1 year from pregnancy and lactation) is the most frequent malignancy in pregnant women, affecting 1:3000 pregnancies in patients between 32 and 38 years old (Keleher et al., 2002; Gentilini et al., 2005; Barnes and Newman, 2007; Keinan-Boker et al., 2008). Because this cancer is age-related, its incidence is increasing as many women delay childbearing until later in life (Barnes and Newman, 2007; Keinan-Boker et al., 2008). These cancers often display lactating features, which are sometimes also noted in breast tumors that arise outside pregnancy/lactation, and have not yet been molecularly characterized. They are generally triple negative and are diagnosed at late stages, typically having poor prognosis (Barnes and Newman, 2007). It is therefore important to understand the pathogenesis of these cancers and elucidate the molecular mechanisms associated with aberrant cell proliferation and lactating features both within and outside pregnancy and lactation.

Normal self-renewal is regulated by a number of genes, which when deregulated may lead to aberrant cell proliferation. OCT4 is a self-renewal transcription factor (TF) that is found silenced in the vast majority of somatic cells. It has been established that the normal resting breast (from non-pregnant, non-lactating women) as well as cell lines derived from it have very little expression of OCT4 and associated embryonic TFs (Tai et al., 2005; Beltran et al., 2011; Lengerke et al., 2011; Hassiotou et al., 2012; Liu et al., 2012). However, we have recently demonstrated high expression of OCT4 and its associated TFs in the normal lactating breast and in cells isolated from breastmilk (Hassiotou et al., 2012). OCT4 is a key TF, which together with SOX2 and NANOG regulates self-renewal and pluripotency in embryonic stem cells (ESCs) (Boyer et al., 2005; Young, 2011). In addition, ectopic expression of OCT4 and other embryonic TFs (such as SOX2 and KLF4) reprograms adult somatic cells to a self-renewing, undifferentiated pluripotent state, named induced pluripotent stem cell (iPSC) (Sterneckert et al., 2012). In addition to ESCs and iPSCs, an increasing body of evidence demonstrates expression of OCT4 and its associated ESC TFs in various adult organs by rare subpopulations of normal stem cells (Tai et al., 2005; Matthai et al., 2006; Conrad et al., 2008; Kuroda et al., 2010; Arnold et al., 2011; Nakatsugawa et al., 2011; Ratajczak et al., 2011), including the breast both in its resting (Hassiotou et al., 2012; Roy et al., 2013) and lactating state (Hassiotou et al., 2012). The common expression of ESC TFs in the early adult stem cell state and their downregulation upon differentiation suggest molecular similarities between the regulation of ESCs and the cell population associated with repair, regeneration, and remodeling in some adult tissues. At the same time, aberrant upregulation of ESC TFs in certain cancers and association with cancer stem-like cell (CSC) proliferation further reinforce ESC TF involvement in both normal adult tissue development and oncogenesis (Monk and Holding, 2001; Kumar et al., 2012).

Previous work has demonstrated expression of OCT4, SOX2, and NANOG in some breast tumors, particularly triple negative cancers that are poorly differentiated and have poor prognosis (Ben-Porath et al., 2008; Lengerke et al., 2011; Liu et al., 2011, 2012). However, the large heterogeneity and variability in expression both between and within tumor tissues make the association of these genes with breast cancer still obscure (Liu et al., 2011; Leis et al., 2012). Expression of OCT4 in the normal lactating breast prompted us to examine whether OCT4 is also expressed in breast tumors with lactating features and/or associated with pregnancy and lactation. We hypothesized that OCT4 expression is associated with lactating features both in the normal breast during lactation and in breast tumors that display such features.

To address this, we quantified OCT4 expression and subcellular localization by immunohistochemical (IHC) staining in rare specimens of normal human breast during the resting (non-lactating) and lactating stages, and in breast cancers with lactating features. Although minimal OCT4 expression was observed in the normal resting breast, OCT4 was upregulated in the normal lactating breast, with a further increase in expression in breast tumors with lactating features. The majority of OCT4^+^ cells in tumor and normal lactating tissues co-expressed NANOG. To further investigate the association of OCT4 with normal and aberrant breast development, we compared expression of OCT4 and associated ESC genes between hESCs and normal self-renewing stem cells accessed via breastmilk (hBSCs) or available OCT4-overexpressing breast cancer cell line models (OCT4-overexpressing tumorigenic breast cell lines, OTBCs) possessing tumor-initiating features. Although expression levels between ESC genes were similar in both hBSCs and hESCs, OTBCs had imbalanced ESC gene expression, with OCT4 levels being significantly higher than those of SOX2 and NANOG. However, similar to hBSCs and hESCs, OTBCs were able to differentiate both *in vitro* and *in vivo* into cells from all three germ layers, suggesting multi-lineage differentiation potential. Importantly, they were also able to differentiate into functional lactocytes that synthesized milk proteins. These findings highlight OCT4 with its associated embryonic TFs as potential key regulators of normal self-renewal and differentiation in the breast during pregnancy and lactation. Further, they suggest that deregulation of OCT4 expression in the breast may result in malignant transformation of breast cells, acquisition of tumorigenic properties, and lactating differentiation potential.

## Materials and Methods

### Tissues

**Figure 1:**
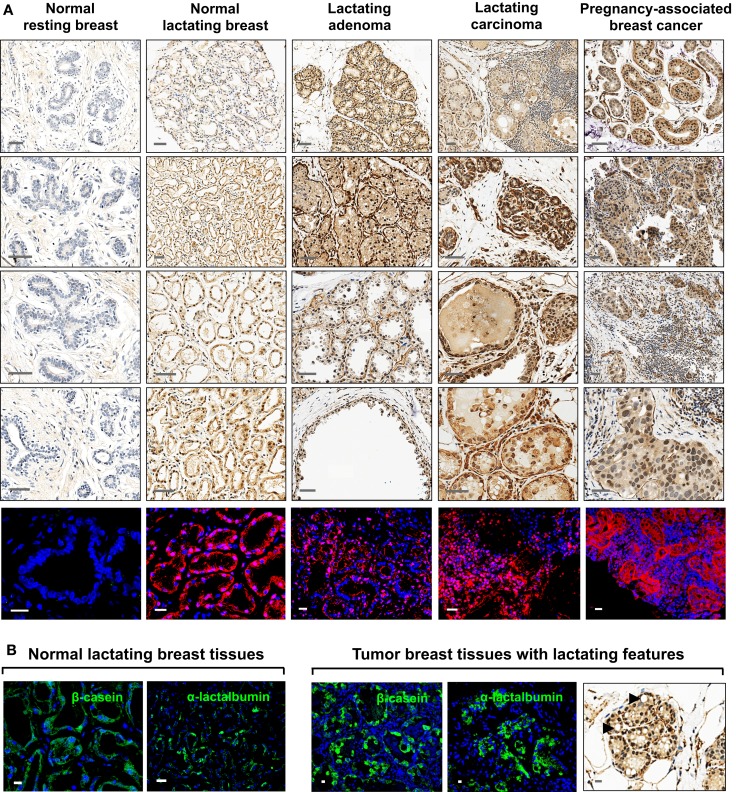
**Expression of OCT4 in the normal resting and lactating breast, and in breast tumors with lactating features or associated with pregnancy and lactation**. **(A)** IHC and IF staining of OCT4. Scale bars: 50 μm (IHC) and 20 μm (IF). Minimal and mostly cytoplasmic expression was seen in the normal resting breast. During normal lactation, OCT4 was upregulated and expressed in both the nucleus and cytoplasm, with varying expression and subcellular localization between different tissues, and between different lobules and alveoli within the same tissue. Enhanced expression was observed in breast tumors with lactating features or associated with pregnancy and lactation. In addition to the epithelium, some expression was seen in the mammary stroma. **(B)** The lactating features of the examined normal lactating and tumor tissues were confirmed via IF staining for milk proteins and the presence of lipid droplets in the epithelium. Scale bars: 10 μm.

All tissues analyzed were biopsied specimens fixed in formalin and embedded in paraffin. Normal human resting (*N* = 2) and lactating (*N* = 6) breast tissues were obtained from the tissue archive of the School of Anatomy, Physiology, and Human Biology, The University of Western Australia. Human breast tumor tissues were obtained from the Tissue Procurement Core Facility of The University of North Carolina Lineberger Comprehensive Cancer Center in accordance with approved institutional review board (IRB) protocols. Tumor tissues were examined by a pathologist and were identified as lactating breast adenomas (*N* = 7), lactating breast carcinomas (*N* = 3; two ductal carcinomas *in situ* grade III from the same patient, and one invasive ductal carcinoma), and PABC (*N* = 1). The tumor tissues were assigned lactating features by the pathologist based on the presence of lipid droplets characteristic of lactation. This was confirmed by immunofluorescence (IF) staining for milk proteins both in the tumors and in the normal lactating breast specimens (Figure 1). Positive controls for comparison of ESC gene expression were seminoma specimens obtained from the Tissue Procurement Core Facility of The University of North Carolina Lineberger Comprehensive Cancer Center in accordance with approved IRB protocols. Human tumors formed in nude/SCID mice in a xenograft assay from OTBCs (Beltran et al., 2011) were used for IF staining for lactating features and multi-lineage differentiation.

### Immunohistochemistry

Sections of 5-μm thickness were prepared for IHC analysis. IHC was carried out in the Bond Autostainer (Leica Microsystems Inc., Norwell, MA, USA 02061). Slides were deparaffinized in Bond Dewax solution (AR9222) and hydrated in Bond Wash solution (AR9590). Heat induced epitope retrieval was performed at 100°C for 30 min in Bond-Epitope Retrieval solution 1 pH 6.0 (AR9961) and blocked with Dako Serum-Free Ready-To-Use Protein Block for 10 min (X0909). Slides were incubated with anti-OCT4 antibody (Table 1) for 2 h. Appropriate negative controls were also prepared. Antibody detection was performed using the Bond Polymer Refine Detection System (DS9800). Slides were counterstained with hematoxylin. Stained sections were dehydrated in a graded alcohol series, coverslipped, and imaged using the Aperio Scanscope XT (Aperio Technologies, Vista, CA, USA 92081). Seminoma tumors were used as positive control for staining quality and subcellular localization. Images were examined using the Spectrum digital pathology platform.

**Table 1:** **Markers examined**.

Marker	Company	Cat. no	Applications used
OCT4	Miltenyi (Stemgent)	130-095-635	FACS (1:100), IF (1:100)
OCT4	Abcam	ab18976	IHC (1:100-1:200), IF (1:100)
NANOG	Santa Cruz Biotech	sc-33759	FACS (1:100), IF (1:100)
NANOG	Abcam	ab80892	IF (1:100)
α-SMA	Sigma-Aldrich	A2547-0.2ML	IHC (1:100)
CK14	Sapphire Bioscience	GTX104124	IF (1:200)
CK18	Abcam	ab32118	IF (1:100)
CK19	Thermo Scientific	MA1-19059	IF (1:100)
EPCAM	Exbio	11-581-C100	IF (1:300)
β-Casein	Santa Cruz Biotech	sc-53189	IF (1:100)
α-Lactalbumin	Dako	A057901	IF (1:1000)
β-III-tubulin	Covance	PRB-435P	IF (1:1000)
Nestin	Miltenyi (Stemgent)	130-095-648	IF (1:200)
Vimentin	Sigma-Aldrich	V5255	IF (1:100)
Desmin	Sigma-Aldrich	D1033-0.2ML	IF (1:100)
Albumin	Sigma-Aldrich	A-3293	IF (1:200)
OV6	R&D Systems	MAB2020	IF (1:200)
PDX1	Santa Cruz Biotech	sc-14662	IF (1:100)
Insulin	Santa Cruz Biotech	sc-52040	IF (1:100)
Insulin	Cell Signalling Tech	4590S	IF (1:100)
C-peptide	Cell Signalling Tech	4593S	IF (1:100)
RUNX2	Santa Cruz Biotech	sc-101145	IF (1:100)
OSX	Santa Cruz Biotech	sc-133871	IF (1:100)
Cardiac T-troponin	Abcam	ab45932	IF (1:100)

*FACS, fluorescence activated cell sorting; IF, immunofluorescence; IHC, Immunohistochemistry*.

### Image quantification

**Figure 2:**
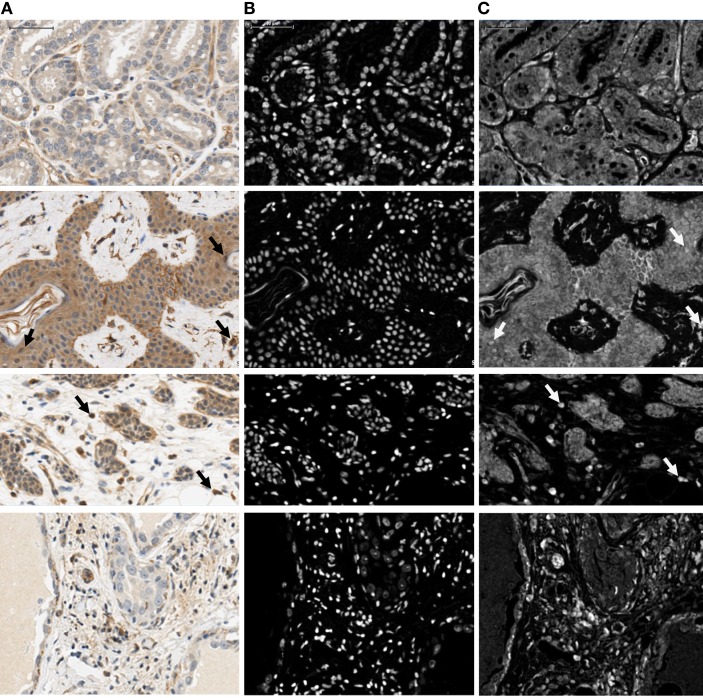
**Illustration showing part of the four image regions used for optimization of nuclear and cytoplasmic sizes, shapes, and IHC thresholds**. Regions were chosen to include the range of cytoplasmic and nuclear intensities that were present in the complete image data set. Raw images **(A)** and corresponding nuclear [hematoxylin; **(B)**] and IHC [DAB; **(C)**] stained layers are shown. Arrows indicate examples of positive nuclei. Scale bar: 50 μm.

**Figure 3:**
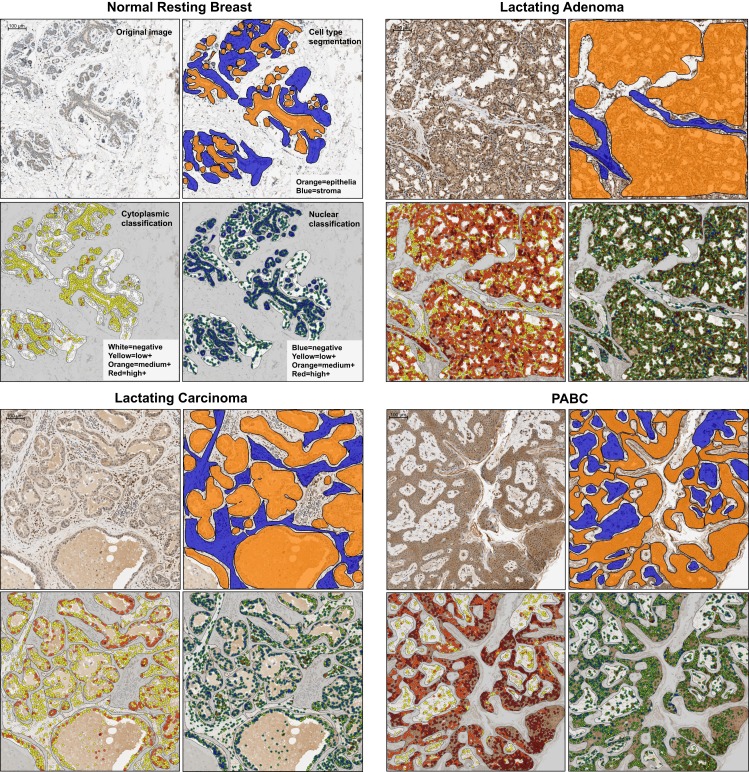
**Representative examples of the examined tissues showing cell type segmentation and cytoplasmic and nuclear classification for different staining intensities used for quantification of OCT4 expression in the epithelium and in the stroma**. For each sample type, the top left panel is the raw image; the top right illustrates the epithelial/stromal segmentation with orange representing epithelial regions and blue the stromal regions; the bottom left panel shows the cytoplasmic classification, with white: negative cytoplasm, yellow: low positive cytoplasm, orange: medium positive cytoplasm, red: highly positive cytoplasm; the bottom right panel shows the nuclear classification, with blue: negative nucleus, yellow: low positive nucleus, orange: medium positive nucleus, red: highly positive nucleus. PABC: pregnancy-associated breast cancer. Scale bars: 100 μm.

Image analysis was performed using the “Nuclei and Simulated Cells” algorithm found in Definiens Tissue Studio software (version 3.5; Munich, Germany). OCT4 expression was quantified in two to five 0.9-μm^2^ squares per image, which were positioned in areas dominated by abnormal tissue. Epithelial and stromal cells were digitally separated using a combination of Tissue Studio Composer, an automated approach for Region of Interest Selection (http://tissuestudio.definiens.com/composer.html), and manual annotations. A first separation using Tissue Studio Composer was followed by inspection of the annotation results and manual adjustment of annotation using Manual Region of Interest Selection for regions where the automated approach did not yield appropriate annotation. The cellular analysis was identical for the squares where Composer was used and those that were hand annotated. For each square, expression in epithelial versus stromal cells was quantified, including information on positiveness (either expression or no expression), subcellular localization (nuclear, cytoplasmic, or both), and intensity of positive expression (low, medium, or high). This was done via a cell scoring system that grouped each cytoplasm into an intensity group (0, 1, 2, 3) and each nucleus into an intensity group (100, 110, 120, 130). Then, the two numbers were added together to give a unique score based on the resulting sum (Table 2). For intensity measurements, positive/negative thresholds were determined by visually evaluating the above background intensity in a test group of images. The test set had a range of cytoplasmic and nuclear DAB intensities. The thresholds for medium and high intensity were set at equal interval steps from the positive/negative. The separation of cytoplasms and nuclei was based on thresholds that were determined for cytoplasms as 0.1 (minimum threshold), 0.3 (low to medium threshold), and 0.5 (medium to high threshold) (Figure 2). For nuclei, compensation for the presence of hematoxylin and of a general brown background haze was done by increasing the baseline to 0.5 as the minimum threshold, and 0.7 and 0.9 as the thresholds for medium and high intensity, respectively. Example screenshots for this analysis are shown in Figure 3. Results were compiled using Microsoft Excel (version 2010).

**Table 2:** **Scoring system for immunohistochemical quantification of OCT4 expression in the breast tissues examined**.

Cell score	Nuclear status	Cytoplasmic status
100	Negative	Negative
101	Negative	Low positive
102	Negative	Medium positive
103	Negative	High positive
110	Low positive	Negative
111	Low positive	Low positive
112	Low positive	Medium positive
113	Low positive	High positive
120	Medium positive	Negative
121	Medium positive	Low positive
122	Medium positive	Medium positive
123	Medium positive	High positive
130	High positive	Negative
131	High positive	Low positive
132	High positive	Medium positive
133	High positive	High positive

### Immunofluorescence

Immunofluorescence staining was done as described in Hassiotou et al. (2012). Antibodies for ESC markers were standardized in human fibroblasts (negative control cells). Briefly, adherent cells were fixed in 1.5% paraformaraldehyde/0.7% sucrose in PBS, followed by permeabilization in 0.1% Triton X in PBS and incubation with primary (Table 1) and secondary antibodies (Alexa Fluor 488, 546, or 555 nm). hBSC and OTBC spheroids were fixed in 3% formaldehyde in PBS, permeabilized in 0.1% PBS-Triton X 100, washed in PBS, and stained with primary (Table 1) and secondary (as above) antibodies. Five-micrometer thick sections of human breast tissues and tumor tissues from the xenograft assays were rehydrated in deionized water and incubated in PBS prior to permeabilization in 0.1% PBS-Triton X 100 and overnight incubation with primary antibody (Table 1) in a humid chamber. Washing in PBS and secondary antibody incubation for 2 h was followed by a final wash and mounting (Dako). Imaging was standardized using negative controls and was done in a Nikon Eclipse Ti inverted optical microscope, a Nikon 90i upright optical microscope, or a Leica DMIRB Inverted Fluorescence/DIC microscope.

### Breastmilk collection and cell isolation

The study protocol was approved by the Human Research Ethics Office of The University of Western Australia. Breastmilk was sourced from healthy breastfeeding women located in Perth, WA, Australia. All participants (*N* = 31) provided informed written consent and expressed mature breastmilk (5–200 ml) on one or more occasions under aseptic conditions using a Medela Symphony pump (Medela AG, Switzerland). Freshly expressed breastmilk was diluted with equal volume of sterile PBS (pH 7.4, Gibco, USA). After centrifugation at 805 × *g* for 20 min at 20°C, the cell pellet was separated from the fat and liquid part skim milk, washed three times in PBS, and was then resuspended in 7% Fetal Bovine Serum (FBS, Certified, Invitrogen, USA) in PBS (blocking buffer). Total breastmilk cell content and viability were measured using a Neubauer hemocytometer by Trypan Blue exclusion.

### Flow activated cell sorting

*Ex vivo* flow activated cell sorting (FACS) analysis of OCT4 and NANOG expression in freshly isolated breastmilk cells was done as described in Hassiotou et al. (2012). Antibodies against these markers (Table 1) were standardized in human fibroblasts (negative control) and were shown to recognize their target proteins by FACS (Stemgent, USA; Santa Cruz Biotechnology, USA). All incubations and washes were done in 0.05% Tween-20 in PBS after initial cell fixation in 1% paraformaraldehyde/0.7% sucrose in PBS for 15 min. Primary antibody incubation was done for 30 min at 4°C followed by secondary antibody incubation (AlexaFluor 488 nm for OCT4 and 647 nm for NANOG; Invitrogen, USA) for 30 min at 4°C at 1:300, final washing and suspension into fixative. Appropriate secondary only negative internal controls were used. Data acquisition was done with a FACS Calibur Flow Cytometer (Becton Dickinson, NJ, USA) and data analysis using FlowJo.

### Cell lines and cell culture

OCT4-overexpressing tumorigenic breast cell lines were used from Beltran et al. (2011) and cultured for examination of multi-lineage differentiation as described for hBSCs by Hassiotou et al. (2012). Freshly isolated breastmilk cells were cultured in spheroid conditions as described previously (Hassiotou et al., 2012).

### Statistical analyses

Statistical analyses were performed in Microsoft Excel and in R 2.9.0 1 for Mac OSX (R Development Core Team, 2009). The results are presented as range and mean ± SD. Ordinary least squares regression models were used to determine whether levels of expression differed between tissues types, with the percentage of cells expressing OCT4 at the specified level as the response and the tissue type classification as the predictor. Level of expression was defined as the proportion of cells in each sample with positive expression of the given level (low, medium, high). Expression for nucleus and cytoplasm was considered separately for each of epithelial and stromal tissue. Tissues of the same type (normal resting breast, normal lactating breast, lactating adenoma, lactating carcinoma, PABC) were grouped and comparisons were made separately with respect to either the normal resting or the normal lactating breast. *P* values <0.05 were considered statistically significant.

## Results

### OCT4 and NANOG are expressed in the normal lactating breast and in breast tumors with lactating features

**Figure 4:**
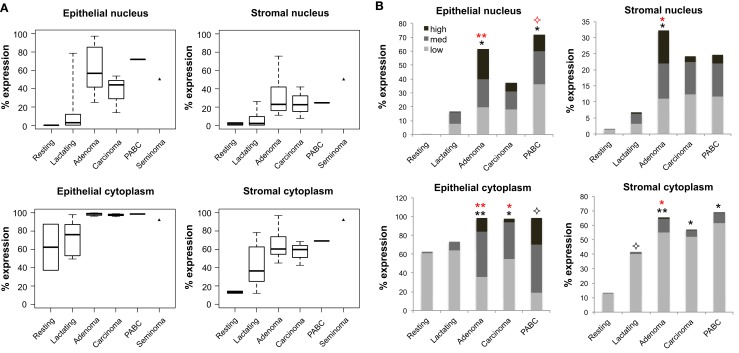
**Quantification of OCT4 expression in the epithelium and stroma of normal and tumor breast tissues**. **(A)** Levels of expression in the nucleus and cytoplasm between tissue types. Boxes show first and third quartiles, horizontal bars within boxes indicate median values, and “whiskers” show the range of values. Seminoma was used as positive control. **(B)** Column charts showing contribution of the different expression levels (low, medium, and high) to the total expression for each tissue type. Significance is shown with stars, whereby black stars compare the respective tissue with the normal resting breast and red stars with the normal lactating breast. Normal human resting breast: *N* = 2; normal human lactating breast: *N* = 6; lactating breast adenomas: *N* = 7; lactating breast carcinomas: *N* = 3; pregnancy-associated breast cancer (PABC): *N* = 1. *P* values for overall levels of expression were determined with OLS regression separately for each location and comparison tissue, and are shown as: 0.05 < 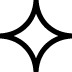 ≤ 0.1; 0.01 < * ≤ 0.05; and 0.001 < ** ≤ 0.01.

**Figure 5:**
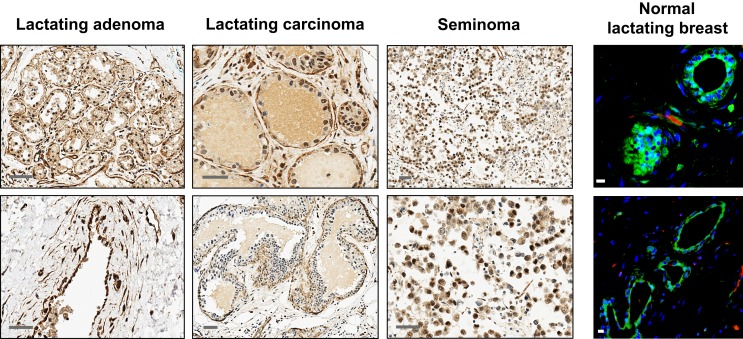
**Additional examples of OCT4 expression by IHC and IF in the normal lactating and breast tumor tissues examined**. Seminomas were used as positive control. Scale bars: 50 μm (IHC), and 10 μm (IF).

Expression of OCT4 was examined by IHC and IF in the normal breast during different stages of development (resting and lactating) and in breast tumors that display lactating features and/or are associated with pregnancy and lactation (Figure 1A). The lactating properties of the examined tissues were confirmed via IF staining for milk proteins and the presence of lipid droplets in the epithelium (Figure 1B). OCT4 expression was detected both in the epithelium and in the stroma, and typically higher expression levels in the epithelium corresponded with higher expression levels in the stroma. However, stromal expression was generally lower than that of the epithelium (Table 3; Figure 1). Levels of expression varied within and between sample types, with different proportions of cells expressing OCT4 in the nucleus, the cytoplasm, or both, and at various intensities, which were classified as low, medium, or high (Figure 4). Despite these intra- and inter-specimen variations, clear trends could be observed for each tissue type (Figure 4). Minimal, and mostly low cytoplasmic, expression was observed in the normal resting breast (range 0.07–0.09%; mean 0.08 ± 0.02% SD nuclear expression), whilst an upregulation in both total (range 32.6–88.5%; mean 57.7 ± 21.5% SD) and nuclear (range 0.2–52.4%; mean 11.4 ± 20.5% SD) expression was seen during normal lactation (Figures 1, 4, and 5; Table 3), with large variation observed between different normal lactating breast tissues. Of this, the highest expression was in the epithelium (range 0.1–78.7%; mean 16.1 ± 31% SD nuclear expression), but positive cells were also present in the stroma (range 0.3–26.1%; mean 6.8 ± 10.1% SD nuclear expression). OCT4 was localized both in the ductal and alveolar lactating epithelium, and was scanty in both the basal and luminal cell layers.

**Table 3:** **Mean levels and standard deviations (SD) of OCT4 expression in the tissues examined**.

Nucleus	Epithelial positive (out of total epithelial)		Stromal positive (out of total stromal)		Total positive	
	Mean (%)	SD (%)	Mean (%)	SD (%)	Mean	SD
Resting breast (2)	0.1	0.0	1.7	2.0	0.9	1.0
Lactating breast (6)	16.1	31.0	6.8	10.1	11.4	20.5
Lactating carcinoma (3)	37.3	20.9	24.1	17.2	30.7	17.4
Lactating adenoma (7)	61.9	28.6	32.3	24.0	47.1	25.1
PABC (1)	72.0	–	24.7	–	48.3	–

**Cytoplasm**	**Mean (%)**	**SD (%)**	**Mean (%)**	**SD (%)**	**Mean (%)**	**SD (%)**

Resting breast (2)	62.4	35.6	13.1	1.7	37.7	16.9
Lactating breast (6)	79.0	17.4	41.7	24.5	57.5	21.4
Lactating carcinoma (3)	97.6	1.7	56.9	13.2	77.3	6.7
Lactating adenoma (7)	98.4	1.4	65.5	18.0	81.9	9.5
PABC (1)	98.6	–	69.1	–	83.9	–

*Percentage positive epithelial cells (out of total epithelial cells), % positive stromal cells (out of total stromal cells), and % total positive cells were determined separately for each sample. For each tissue type, *N* is shown in parentheses. Nuclear and cytoplasmic expressions are shown separately*.

**Figure 6:**
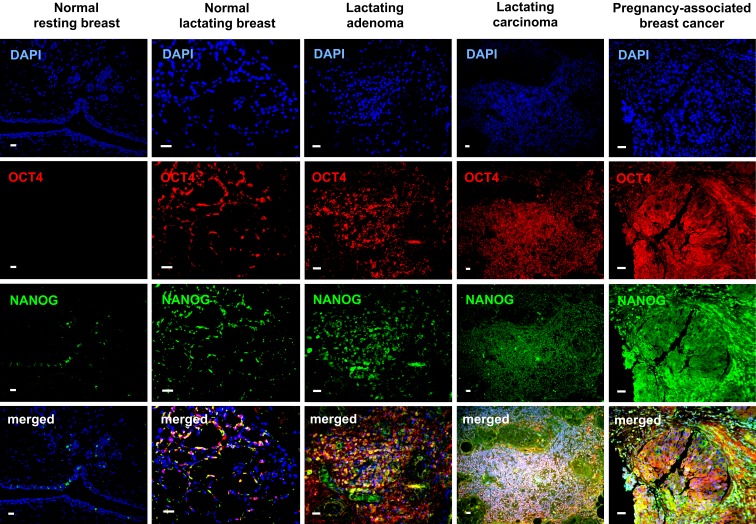
**Co-localization of OCT4 (red) and NANOG (green) in the normal lactating breast and in breast tumors with lactating features or associated with pregnancy and lactation**. Scale bars: 20 μm. Blue: DAPI stain for nuclei.

OCT4 nuclear expression was further upregulated in the breast tumors examined, with the lactating adenomas showing the highest upregulation compared with the normal resting (*P* = 0.011) and lactating breast (*P* = 0.007) (Table 3; Figure 4). Of note, stronger expression in the epithelium of lactating adenomas was observed in the basal layer compared with the luminal layer (Figure 1). The PABC case examined showed one of the highest OCT4 nuclear expressions, with marginally significant differences with the normal resting (*P* = 0.043) and lactating breast (*P* = 0.07) (Table 3; Figure 4). Significant increases in cytoplasmic OCT4 expression were also seen between the breast tumor tissues and the normal resting (*P* = 0.008 for lactating adenomas; *P* = 0.019 for lactating carcinoma) and lactating breast tissues (*P* = 0.008 for lactating adenomas; *P* = 0.033 for lactating carcinoma), with the highest expression observed in lactating adenomas (Table 3; Figure 4). The increase in cytoplasmic expression in PABC was marginally significant compared with the normal resting breast (*P* = 0.061). OCT4 was co-localized with NANOG in the majority of positive cells (Figure 6). In the normal resting breast tissues, expression of NANOG was somewhat higher than that of OCT4, but an upregulation was observed for both TFs in the normal lactating breast (Figure 6). Similarly to OCT4, NANOG was also upregulated in the tumor tissues examined and showed a pattern of expression similar to OCT4 (Figure 6).

### Differential expression of OCT4 and associated ESC genes between normal hBSCs and OCT4-transduced tumorigenic cells

**Figure 7:**
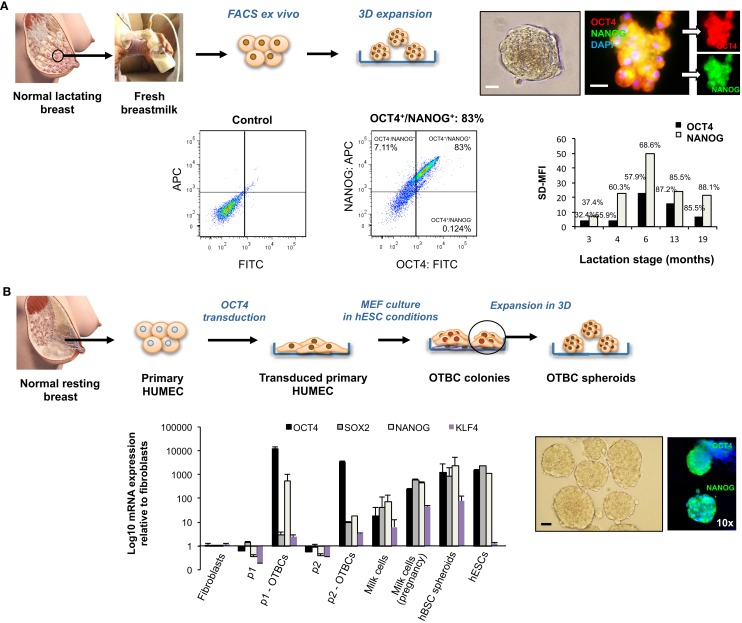
**Embryonic stem cell transcription factor expression and effects on self-renewal of normal stem cells from the lactating breast and OCT4-overexpressing cancer cells (OTBCs)**. **(A)** Normal stem cells of the lactating breast accessed via breastmilk. The panel shows FACS *ex vivo* analysis of OCT4 and NANOG co-expression in freshly isolated breastmilk cells, and a column chart of the standardized difference in Mean Fluorescence Intensity between the test and the control (SD-MFI), and the% positive cells for each sample (*N* = 5), each of which represents a different lactation stage. In addition, hBSC spheroids obtained in 3D culture that co-express OCT4 and NANOG are shown. Blue: DAPI nuclear stain. Scale bars: 20 μm. **(B)** The Workflow of derivation of OCT4-transduced cells (OTBCs) from resting breast cells is shown (adapted from Beltran et al., 2011). The IF images present OTBC spheroids grown in 3D culture expressing OCT4 and NANOG. Blue: DAPI nuclear stain. The column chart compares mRNA expression levels of OCT4, SOX2, NANOG, and KLF4 among parental lines (p1 and p2), OTBC lines (p1-OTBCs, *N* = 6; and p2-OTBCs, *N* = 2, respectively), fresh milk cells (*N* = 16), fresh milk cells during pregnancy [“Milk cells (pregnancy),” *N* = 1], hBSC spheroids (*N* = 10–31, depending on the gene), fibroblasts, and hESCs (adapted from Beltran et al., 2011; Hassiotou et al., 2012). Individual PCR reactions were normalized against GAPDH and plotted relative to the expression level of fibroblasts. Bars represent the mean ± SEM for fibroblasts, parental lines, p2-OTBCs, milk cells-P, and hESCs, and the mean ± SD for p1-OTBCs, milk cells, and hBSC spheroids. Images of normal lactating and resting breasts: ©Medela AG, Switzerland, 2006. Used with Permission.

To further examine differences in expression of OCT4 and associated ESC TFs between normal lactating breast stem cells and breast CSCs, we took advantage of breastmilk as a source of stem cells from the normal lactating breast, and of OCT4-overexpressing breast cancer cell line models (OTBCs), given that cell lines isolated from tumors that have high OCT4 expression are not commercially available. Breastmilk cells were characterized *ex vivo* by FACS for co-expression of OCT4 and NANOG. Mostly, these TFs were co-expressed and levels of expression ranged 32–88% of total breastmilk cells (Figure 7A), which is very similar to the total expression levels observed in the normal lactating breast tissues (Figure 4). An effect of lactation stage could be seen on OCT4/NANOG protein expression in a cross-sectional dataset of breastmilk samples, with a peak expression at 6 months postpartum, and a decrease in expression in later lactation (Figure 7A). In spheroid culture conditions, hBSCs rapidly proliferated to form spheroids that increased in size in a time-dependent manner and co-expressed OCT4 and NANOG (Figure 7A). RT-PCR analysis of mRNA expression of the ESC TF circuitry (OCT4, SOX2, and NANOG) demonstrated an upregulation of these genes in fresh breastmilk cells compared with resting breast cells and fibroblasts, with the highest expression observed at pregnancy concurrent with lactation (Figure 7B). Expression was further increased in hBSC spheroids, which reached levels similar to hESCs (Figure 7B) (Hassiotou et al., 2012).

OCT4-overexpressing tumorigenic breast cell lines acquired a highly proliferative character compared with their parental cells, which was enhanced in spheroid conditions (Figure 7B). Concurrent with OCT4, they showed upregulation of NANOG and to a lesser degree of SOX2 (Figure 7B). However, both the absolute and relative levels of ESC TF expression were markedly different in OTBCs compared with hBSCs or hESCs. OCT4 mRNA expression levels in OTBCs were 2–10 times higher than hESCs and breastmilk spheroids, and 26–95 times higher than fresh breastmilk cells (Figure 7B). SOX2 expression was markedly lower in OTBCs than hESCs (230–753 times lower) or breastmilk-derived cells (33–281 times lower) (Figure 7B). NANOG expression was also lower in OTBCs than hESCs (2–64 times lower) or breastmilk-derived cells (4–130 times lower) (Figure 7B). These findings suggest differential expression of the ESC TF circuitry between normal breast stem cells and breast CSCs.

### OCT4-transduced cells have multi-lineage differentiation potential

**Figure 8:**
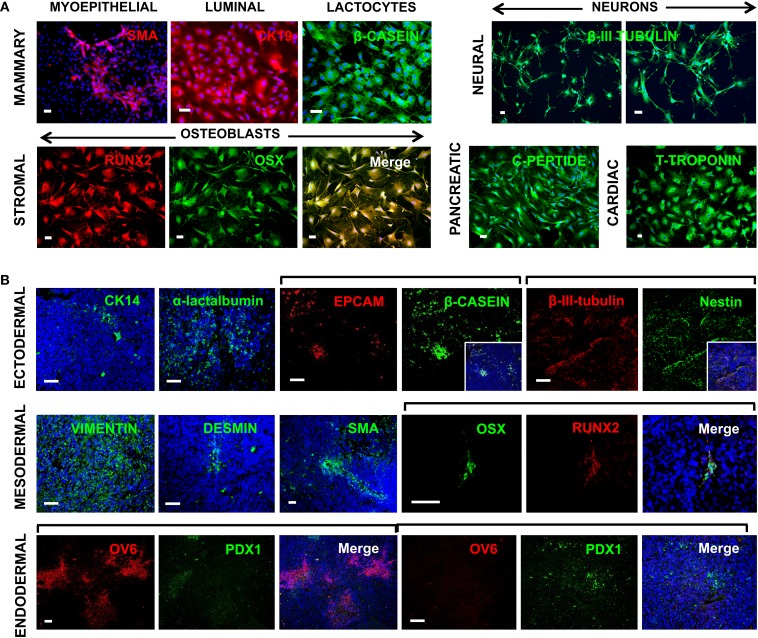
**Multi-lineage differentiation capabilities of OCT4-overexpressing cancer cells (OTBCs)**. **(A)** OTBCs directed to differentiate *in vitro* into myoepithelial SMA^+^ cells, luminal CK19^+^ cells, β-casein^+^ lactocyte-like cells, β-III-tubulin^+^ neuron-like cells, RUNX2^+^/OSX^+^ osteoblast-like cells, c-peptide^+^ islet-like cells, and T-troponin^+^ cardiomyocyte-like cells. **(B)** Tumors formed in nude/SCID mice by OTBCs contained cells from all three germ layers. Blue: DAPI nuclear stain. Scale bars: 50 μm.

Given the function of OCT4 and its associated ESC TF circuitry in controlling the pluripotential of hESCs (Young, 2011), it is of interest to examine whether expression of OCT4 by breast cells is associated with multi-lineage differentiation properties. We have previously demonstrated that hBSCs can differentiate into cells from all three germ layers both spontaneously and under directed differentiation in specific microenvironments (Hassiotou et al., 2012). Here, we examined the potential of OTBCs to differentiate into mammary and non-mammary lineages. Similarly to hBSCs, OTBCs were able to differentiate into mammary ductal luminal CK19^+^ cells, SMA^+^ myoepithelial cells, and lactocytes that produced β-casein under mammary differentiation conditions. Furthermore, they also differentiated into cells from non-mammary lineages, including β-III-tubulin neuron-like cells, RUNX2^+^/OSX^+^ osteoblast-like cells, c-peptide-producing islet-like cells, and T-troponin^+^ cardiomyocyte-like cells (Figure 8A). Of note, due to the propagation of OTBCs in spheroid culture, later passages acquired a migratory character and were less adhesive; hence differentiation efficiency was greater in earlier passages of OTBCs rather than in later passages. In addition, we examined multi-lineage differentiation of OTBCs in the tumors that these cells generated when injected in nude/SCID mice. Despite the relatively poor differentiation state of these tumors, areas with differentiated morphology and phenotype were detected via staining for lineage-specific markers (Figure 8B). These included epithelial markers such as CK14 and EPCAM, with some cells being positive for milk proteins, suggesting lactating features (Figure 8B). The neural markers nestin and β-III-tubulin were also expressed (Figure 8B). Mesodermal markers such as vimentin, desmin, SMA, and the osteoblast phenotype RUNX2/OSX were expressed (Figure 8B). Endodermal lineage cells were also present, including cells expressing the endodermal progenitor marker OV6 and the islet marker PDX1 (Figure 8B). In addition, areas with the distinct phenotypes OV6^high^/PDX1^−/low^ and OV6^−/low^/PDX1^high^ were detected (Figure 8B), suggesting different stages of differentiation within a tumor. These results suggest that expression of OCT4, either normal or aberrant, is associated with multi-lineage differentiation potential and conferral of lactating features.

## Discussion

**Figure 9:**
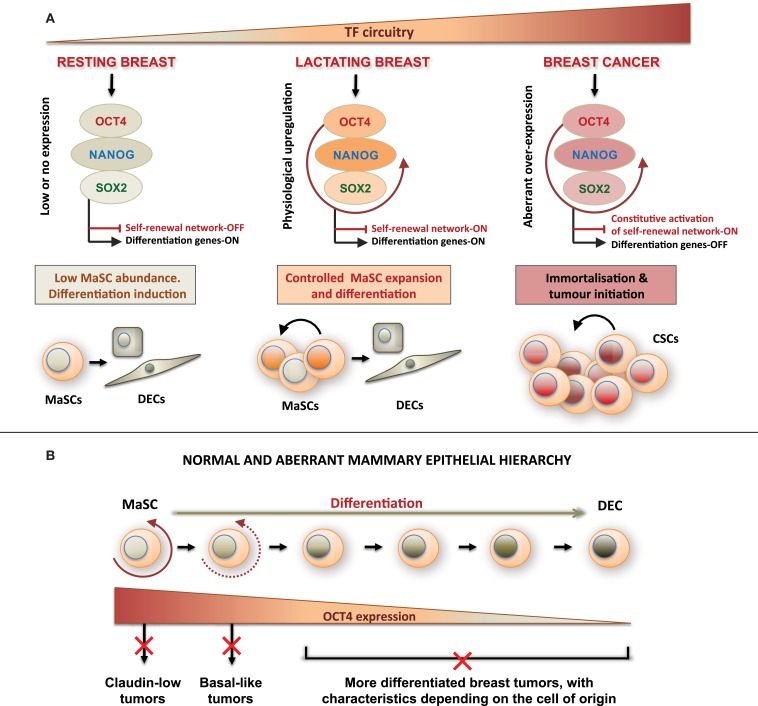
**Proposed model of mammary cellular hierarchy integrating expression of OCT4 and its associated embryonic stem cell gene network in the regulation of self-renewal, differentiation, and transformation in the breast**. **(A)** The resting breast is characterized by minimal expression of the ESC TF circuitry, which allows maintenance of quiescence and low self-renewal activity in MaSCs. During pregnancy and lactation and under physiologic cues, OCT4 promoter becomes activated effecting transient activation of other ESC TFs and downstream targets. This regulates a controlled program of MaSC expansion and subsequent differentiation. Uncontrolled overexpression of OCT4 and/or its associated ESC TFs leads to oncogenic activation of TFs, transformation, and aberrant expansion of the target cell, which may be a MaSC or a more differentiated cell, and acquisition of a CSC phenotype. **(B)** Normal and aberrant mammary epithelial hierarchy based on changing expression of OCT4 that determines the cell state in a mammary developmental continuum. Deregulation of OCT4 expression at each developmental state results in cell transformation and generation of different tumor subtypes. DECs, differentiated epithelial cells; CSCs, cancer stem cells.

Molecular determinants of lactation features and aberrant cell proliferation by some breast tumors are not well understood. Here, we confirm and extend previous findings of expression of ESC self-renewal TFs in both normal lactation and breast cancer. We have previously reported upregulation of OCT4 and its associated ESC genes in the normal lactating breast and breastmilk-derived stem cells (Hassiotou et al., 2012). This prompted us to examine expression of OCT4 in breast tumors with lactating features and compare it with the normal breast. We found that OCT4 is physiologically upregulated during lactation compared with the resting breast, suggesting a potential role of this gene and its associated ESC gene network in the normal mammary stem cell (MaSC) self-renewal that fuels the remodeling of the breast into a fully mature milk-secretory organ. The association of OCT4 with lactation was further demonstrated by its aberrant expression in breast tumors with lactating features, in which expression levels were significantly higher than in the normal breast (*P* < 0.01). Some of these tumors may be associated with pregnancy and lactation, although further research is needed to underpin the molecular characteristics of these tumors. Comparison of normal hBSCs with tumorigenic OCT4-tranduced cells showed differential expression of ESC TFs in the two systems. We propose that the levels and balance of expression of ESC TFs in breast cells distinguish between a normal and a CSC (Figure 9). Expression of OCT4 in both normal and CSCs appears to be associated with lactation features and multi-lineage differentiation capabilities, in accordance with the role of these TFs in maintaining pluripotency in ESCs and iPSCs.

A unifying feature between the normal lactating breast and the examined breast tumors was upregulation of OCT4, which was co-localized with NANOG in the majority of positive cells. In contrast to the normal resting breast, where expression of OCT4 and NANOG were minimal, mostly in cytoplasms and at low levels, lactating features both in the normal and transformed breast were associated with upregulation of these TFs, particularly in the nucleus. Cytoplasmic staining for ESC TFs in the breast has also been reported previously (Lengerke et al., 2011), and may be suggestive of either non-specific/background staining or true cytoplasmic expression indicative of a different function, isoform, and/or transcriptional/post-transcriptional regulation of these genes in the cytoplasm. The latter is further supported by the shift toward nuclear expression in the lactating tissues, and merits further investigation.

Significant variation in OCT4 expression was observed among the normal lactating breast tissues examined, which is in agreement with the variable expression levels detected in breastmilk-derived cells (Hassiotou et al., 2012). Here, we show some evidence that this variation may be related to the stage of lactation (Figure 7A). Moreover, differential expression was seen within each tissue, with some lobules and alveoli showing higher expression than others. This demonstrates a heterogeneity in cell differentiation and maturation between and within different lobules within a breast, as has been reported for other species (Molenaar et al., 1992), and suggests that variations observed between breastmilk samples may at least in part reflect emptying of specific lobules during the milk expression process.

In agreement with the association of these TFs with lactation, tumors with lactating features showed upregulation of OCT4, but with much higher expression than the normal lactating epithelium (*P* < 0.05), often concentrated in the basal layer. This suggests that OCT4 confers a tumorigenic character when expressed at levels higher than the normal range. In support of this, forced ectopic expression of OCT4 in resting breast cells produced cells with high proliferation capacity that resembled the claudin-low subtype, and which formed primary tumors and metastases in nude/SCID mice (Beltran et al., 2011). By contrast, hBSCs, which originate from the normal lactating breast, did not form tumors when injected subcutaneously in SCID mice (Hassiotou et al., 2012). When compared with hBSCs and hESCs, OTBCs had markedly higher expression of OCT4 and lower expression of SOX2 and NANOG (Figure 7B). These TFs were expressed at similar levels in each of hESCs and cells from the normal lactating breast, but this balance was completely deregulated in OTBCs (Figure 7B).

Taken together, these results suggest that the distinguishing factor between normal MaSCs and breast CSCs may be the balance between and expression levels of TFs, which control breast cell self-renewal. These findings are integrated in Figure 9A, which proposes a model by which expression levels of the pluripotency TF circuitry control MaSC self-renewal, differentiation, and aberrant transformation. At the same time, regulation of other TFs associated with these genes as well as their downstream targets may contribute to the normal versus aberrant stem cell self-renewal. To this end, two recent studies reported significantly lower disease-free survival in patients with strong OCT4 and NANOG expression, and better survival rates in patients with high KLF4 expression (Liu et al., 2011; Nagata et al., 2012). KLF4 acts in concert with OCT4, SOX2, and NANOG in hESCs (Young, 2011), but is downregulated in some breast cancer cells compared with normal mammary cells (Akaogi et al., 2009). Interestingly, KLF4 expression was higher in breastmilk-derived cells than in OTBCs and hESCs (Figure 7D). This may in part explain the non-tumorigenic character of normal MaSCs and of hBSCs (Hassiotou et al., 2012) in contrast to OTBCs and hESC lines (Lensch et al., 2007; Beltran et al., 2011), suggesting that high KLF4 expression may be a protective factor against aberrant cell proliferation during normal lactation.

In addition to self-renewal, OCT4, SOX2, and NANOG control pluripotency in hESCs and iPSCs (Takahashi et al., 2007; Young, 2011). This prompted us to examine whether hBSCs and OTBCs, which both express these genes, have multi-lineage differentiation potential. Indeed, we have previously shown that hBSCs can differentiate into cells with properties of functional ectodermally-, mesodermally-, and endodermally-derived cells (Hassiotou et al., 2012). Here, we show that similarly to hBSCs, OTBCs are able to differentiate into cells from the three germ layers, including functional lactocyte-like cells that synthesized milk proteins, and islet-like cells, myoepithelial cells, neuron-like cells, osteoblast-like cells, and cardiomyocyte-like cells, with phenotype and morphology of the corresponding cell lineages (Figure 8B). Further, OTBC-originated tumors in nude/SCID mice contained cells with phenotypes from different lineages, including cells that synthesized milk proteins (Figure 8B). Of note, only certain cell subpopulations underwent differentiation in these xenograft tumors, while the remaining cells were in a poor differentiation state, possibly due to their high level of OCT4 expression. We propose that expression of this TF pluripotency network, either in normal MaSCs or in transformed MaSCs, confers lactation features under mammary conditions and multi-lineage differentiation capabilities in tissue-specific microenvironments. This notion may provide insight into the origin and properties of metaplastic breast tumors as well as mechanisms behind breast cancer metastasis. The presence of OCT4^+^/NANOG^+^ cells in the mammary stroma both in the normal lactating and the tumor tissues examined (Figures 1 and 4) further supports this theory, suggesting that self-renewal and differentiation in the mammary stroma may be controlled by the same TF network that regulates the mammary epithelium. This gene network may differentially respond to different micro-environmental cues to control lineage-specific differentiation.

To our knowledge, this is the first study that directly compares ESC TF expression and subcellular localization between normal lactation and breast cancer, enabling a first examination of expression differences in these rare specimens. A limitation of the current data is the number of samples available for IHC analysis. However, normal human lactating breast tissue specimens are extremely rare, which is why the normal human lactating breast has been scarcely studied previously. Similarly, although breast tumor tissues with lactating features may not be as rare as previously thought, pathologists do not routinely look for and designate lactating features in tumor samples, rendering the acquisition of such samples problematic. Further studies are needed to confirm and expand the current findings, also including normal breast samples during early, middle and late pregnancy, to improve understanding of ESC TF expression during the normal pregnancy/lactation cycle.

An important outcome of our study was the demonstration of ESC TF expression changes in different developmental stages of the normal adult breast. Previous focus on the resting breast, where MaSCs are scarce (Tiede and Kang, 2011) and these TFs are silenced (Lengerke et al., 2011), has hindered examination of a role of these genes in mammary development. We showed a physiological upregulation of these genes during lactation, opening new avenues for investigation of their function during mammary development, such as associations with lactation efficiency and postpartum involution. At the same time, these TFs may be used as new candidate markers for delineation of the cellular hierarchy of the mammary gland, which has been problematic due to imprecision of the current markers known to identify different mammary subpopulations (Prat and Perou, 2009). Figure 9B proposes a simplified model of normal and aberrant mammary hierarchy based on expression of OCT4. The varying expression and subcellular localization of OCT4 both in the normal lactating breast and in the examined breast tumors together with the relatively small proportion of highly positive cells in the nucleus in all cases suggest that OCT4 is differentially expressed along a differentiation continuum of the mammary cellular hierarchy, with distinct expression levels and subcellular localization in different cell developmental states, and strongest expression in MaSCs. This model gives further insight into the CSC theory, proposing that differential aberrant upregulation of OCT4 and its associated ESC genes together with the developmental state of the targeted cell may explain tumor heterogeneity and the different breast cancer subtypes. This is consistent with previous studies suggesting differential cellular origins of the different breast cancer subtypes (Lim et al., 2009; Prat and Perou, 2009; Thomas et al., 2012). Future studies will utilize breastmilk as a non-invasive and personalized source of the cellular hierarchy of the fully mature gland to study the normal biology of this organ, molecular determinants of breast cancer, and potentially breast cancer risk.

## Conclusion

OCT4 and its associated embryonic TFs emerge as potential regulators of self-renewal and potency in early-stage MaSCs, which are scarce in the resting breast, but are activated to undergo a controlled program of proliferation and differentiation toward milk-secretory cells during pregnancy and lactation. In addition to their role during normal lactation, these genes may control breast oncogenesis by reverting normal mammary cells to a CSC self-renewing phenotype when expressed aberrantly. It is proposed that disruption of this controlled program of gene expression during pregnancy and lactation, failure to appropriately silence these genes during postpartum involution, and/or their aberrant imbalanced upregulation in the resting breast can be at the origin and progression of aggressive breast cancers, which may display lactating features. Future work will utilize the cellular hierarchy of breastmilk as a model to elucidate genetic events that transform cells along the mammary developmental continuum to give rise to different subtypes of breast cancer.

## Author Contributions

Foteini Hassiotou: conception and design, financial support, collection and assembly of data, data analysis and interpretation, manuscript writing, and final approval of manuscript; Anna R. Hepworth: statistical analysis and final approval of manuscript; Adriana S. Beltran: collection of data and final approval of manuscript; Michelle M. Mathews: provision of study material, collection of data, and final approval of manuscript; Alison M. Stuebe: provision of study material and final approval of manuscript; Peter E. Hartmann: financial support, data interpretation, and final approval of manuscript; Luis Filgueira: data interpretation and final approval of manuscript; Pilar Blancafort: conception and design, financial support, data interpretation, and final approval of manuscript.

## Conflict of Interest Statement

The authors declare that the research was conducted in the absence of any commercial or financial relationships that could be construed as a potential conflict of interest.
